# A scalable model for child road safety in low- and middle-income countries: evidence and lessons from the ‘slow zones, safe zones’ intervention in Vietnam

**DOI:** 10.3389/fpubh.2026.1721586

**Published:** 2026-02-24

**Authors:** Cuong Pham, Le Nguyen, Hong Bui, Mirjam Sidik, Phong Le, Atsani Ariobowo

**Affiliations:** 1 Center for Injury Policy and Prevention Research, Hanoi University of Public Health, Hanoi, Vietnam; 2 AIP Foundation, Hanoi, Vietnam; 3 FIA Foundation, London, United Kingdom

**Keywords:** injury prevention, road safety, school zone, sustainable development goals (SDG), Vietnam

## Abstract

**Introduction:**

Road traffic injuries are a leading cause of child mortality in Vietnam, where rapid motorization and the absence of standardized safe school zones create high-risk environments for students. This paper evaluates the “Slow Zones, Safe Zones” (SZSZ) project, a comprehensive, multi-year intervention in Pleiku City designed to reduce road crash injuries and fatalities by making school zones safer.

**Methods:**

The project employed a phased design, beginning with a quasi-experimental pilot study (2018–2020) and expanding to a city-wide, pre-post evaluation (2020–2022). The multi-pronged strategy combined infrastructure upgrades, education and public awareness campaigns, speed limit establishment and enforcement, and policy development. Project effectiveness was evaluated using evidence-based tools, including iRAP Star Rating for Schools (SR4S) assessments, vehicle speed surveys, and student crash and community KAP (Knowledge, Attitude, Practice) surveys.

**Results:**

The intervention yielded statistically significant improvements across all key indicators. iRAP Star Ratings for school zones dramatically improved from as low as 1-star to 4- and 5-star ratings post-intervention. Mean vehicle speeds were reduced by up to 28.9%. The proportion of student crashes occurring within school zones plummeted from 35.6% at baseline to 2.9% at the project’s end. Parental knowledge of correct speed limits increased substantially from 15.6 to 93.2% (*p* < 0.001). The project also catalyzed a legal decision by city authorities formally adopting a “Safe School Zone definition”.

**Discussion:**

The SZSZ project was highly effective, demonstrating that a comprehensive, evidence-based approach can significantly mitigate road risks for children in an LMIC context. Its success in translating local data into official policy provides a powerful, scalable framework for national reform. This model holds significant potential for replication in other low- and middle-income countries, offering a clear pathway to improve child road safety and help achieve the global targets of SDG 3.6.

## 1 Introduction

Road traffic injuries (RTIs) constitute a severe and largely preventable public health crisis, exacting a heavy toll on global mortality and morbidity. Annually, road crashes are responsible for an estimated 1.19 million deaths and cause disabling injuries for millions more. The burden of this crisis is not distributed equally; low- and middle-income countries (LMICs) tragically account for over 90% of all road traffic fatalities, despite possessing far fewer vehicles than high-income nations (1). This disparity underscores a critical global equity issue, where the most vulnerable populations face the greatest risks.

Recognizing the urgency of this epidemic, the international community has integrated road safety into its development agenda through Sustainable Development Goal (SDG) 3.6 (2), which aims to “by 2030, halve the number of global deaths and injuries from road traffic accidents.” Achieving this target requires concerted efforts, particularly in nations experiencing rapid economic and social change.

The burden of road traffic injuries (RTIs) on children and adolescents in Vietnam is a critical public health crisis. As the second leading cause of death for children aged 5–14 and a primary cause of mortality for adolescents, RTIs pose a significant threat to the nation’s youth (3). This alarming trend is exacerbated by Vietnam’s rapid motorization—characterized by a massive increase in motorcycle use—which has occurred alongside the slower development of safe road infrastructure. This high-risk environment renders children, whether as pedestrians, cyclists, or motorcycle passengers, exceptionally vulnerable. The risk of fatal or severe outcomes is significantly amplified by the high incidence of head injuries, a direct consequence of inconsistent helmet use among young riders and passengers (4). This devastating toll on families and the healthcare system highlights an urgent need for targeted interventions in infrastructure, policy, and public awareness to protect the nation’s future generation. The environment around schools presents a concentrated zone of heightened risk. High traffic volumes during peak drop-off and pick-up times, combined with a general lack of traffic calming measures, create a chaotic and hazardous environment for student pedestrians and cyclists. Speeds often remain dangerously high in these areas, and there is a frequent and critical absence of essential pedestrian infrastructure. Many school zones lack clearly marked crosswalks, contiguous sidewalks, traffic signals, and speed bumps, forcing children to navigate directly through active, unpredictable traffic (5, 6).

Furthermore, pervasive poor road user behavior, including illegal parking that obstructs visibility and a common failure among drivers to yield to pedestrians, compounds the danger significantly. A major systemic issue underpinning this problem is the absence of a nationally standardized and mandated “safe school zone” concept in Vietnam. While some individual schools or localities have implemented safety measures on an ad-hoc basis, the approach is inconsistent and not guided by a comprehensive national policy or engineering standard. This systemic gap means that basic safety features that are standard in many other countries are not guaranteed for more than 18.5 million Vietnamese school children, leaving them disproportionately vulnerable on their daily journey to education (7).

In response, the AIP Foundation’s “Slow Zones, Safe Zones” (SZSZ) project was developed as a targeted intervention. This paper aims to evaluate the effectiveness and impact of the SZSZ project across both phases, using the empirical data collected to analyze its outcomes and discuss its implications for achieving national and global road safety targets.

## 2 Methods

### 2.1 Study design

The study employed a sequential, multi-phase mixed-methods design. Phase I (April 2018 – May 2020) utilized a quasi-experimental longitudinal design with concurrent controls to establish the intervention’s effectiveness (proof-of-concept). Two intervention schools (Nguyen Luong Bang Primary and Phan Dang Luu Primary) received the full suite of interventions, while one control school (Anh Hung Nup Primary) received no intervention, allowing for comparison. Data were collected at three key points: baseline (April 2018), mid-line (May 2019), and end-line (May 2020). Phase II (July 2020 – May 2022) transitioned to a city-wide pre-post evaluation design. This shift was predicated on an ethical and practical imperative to scale the intervention to all 31 primary schools in Pleiku City, prioritizing equitable public health impact over the maintenance of a control group. The primary focus was on wider implementation and policy change.

### 2.2 Study setting and population

The study was conducted in Pleiku City, the capital of Gia Lai Province in Vietnam’s Central Highlands. The intervention targeted the city’s entire primary school network, covering 31 schools, approximately 26,632 students, and 1,062 teachers. While all 31 schools received the comprehensive education, policy, and enforcement interventions, the infrastructure impact evaluation focused on 27 schools. The remaining four schools were allocated for upgrades under local government funding cycles; as these physical works were pending completion at the time of evaluation, these sites were excluded from the specific iRAP and speed analysis datasets, though they remained beneficiaries of the broader project activities.

### 2.3 Interventions

The SZSZ project implemented a multi-component intervention strategy:

- Infrastructure: Physical modifications to the road environment included road surface upgrades, construction of new pavements and sidewalks, installation of high-visibility road signs, school zone markings, rumble strips, raised pedestrian crossings, safety railings, and solar-powered flashing lights to alert drivers.
- Education and Awareness: The project launched a multi-faceted road safety initiative featuring a student e-curriculum and educational materials for parents and the community, which was promoted through a wide-reaching communication campaign on social media, national radio, provincial television, and at local events.
- Speed Establishment and Enforcement: The project first worked with local authorities to establish designated school zones with 30–40 km/h speed limits. Subsequently, the project coordinated with local traffic police to strengthen enforcement and ensure compliance within these zones during peak school travel hours.
- Policy Development: A key long-term goal was to effect systemic change. This involved working with government partners to develop an official School Zone Definition and a national Safe School Zone (SSZ) Guide to standardize safety measures across the country.

### 2.4 Data collection methods and instruments

A variety of instruments were used to collect quantitative and qualitative data:

- iRAP Star Rating for Schools (SR4S): This globally recognized tool was used pre- and post-intervention in both phases to provide an objective, evidence-based measure of road infrastructure safety for pedestrians.
- In Phase I, spot-speed data were collected using handheld radar guns. To address the limitations of discontinuous sampling and to capture broader traffic dynamics, Phase II transitioned to a validated video-based traffic analysis method developed by the University of Transport and Communications (UTC). This system utilizes high-angle cameras to continuously record traffic flow, allowing for the precise calculation of mean, 85th percentile (V85), and 15th percentile (V15) speeds, alongside geometric road analysis. While this technological upgrade precludes a direct linear comparison of raw variance between phases, it provides a more robust dataset for the city-wide rollout.
- Speed measurement surveys: In Phase I, vehicle speeds were recorded using handheld speed guns in collaboration with the Pleiku Traffic Police Department. In Phase II, a camera-based system was used to capture vehicle speeds, traffic volume, and road conditions from a high position, a method developed and validated by the Hanoi University of Transport and Communication (UTC).
- Knowledge, Attitude, and Practice (KAP) Surveys: Questionnaires were administered to parents to assess road safety knowledge, attitudes towards speed limits, and self-reported safety practices.
- Student Self-Report Crash Surveys: A structured questionnaire was administered to students to gather data on the incidence and location of road crashes. The recall period for the Phase I baseline survey was the preceding 12 months. To reduce recall bias and enable continuous monitoring during the city-wide rollout, the recall period for Phase II surveys was shortened to the preceding 3 months.

### 2.5 Key variables and indicators

The project’s success was measured against a set of key variables and performance indicators tied to its core objectives:

- Infrastructure safety: The primary variable was the objective safety rating of the road infrastructure. The key indicator was the iRAP Star Rating, with the goal of upgrading school zones from a low (1–2 star) rating to a safer (3-star or higher) global standard.
- Vehicle speed: Speed data was calculated including maximum, minimum, average, and percentile speeds. In particular, the 85th percentile speed (V85) was analyzed, defined as the speed at which 85% of traffic travels at or below.
- Road crash incidents: The key outcome variable was the rate of road crashes involving students. This was measured through the percentage reduction in student self-reported road crashes from baseline to the end-line survey.
- Knowledge, Attitudes, and Practices (KAP): This variable assessed changes in community awareness and behavior. Key indicators were the percentage increase in correct road safety knowledge, the percentage of participants holding positive attitudes towards speed reduction, and the percentage increase in self-reported safe practices among parents.

### 2.6 Data management and analysis

Quantitative data were entered into a database for management, Data analysis and visualization were conducted using R. Descriptive statistics (means, frequencies, percentages) were used to summarize the data. Chi-squared tests were used to assess the statistical significance of changes in categorical data (e.g., KAP survey responses), while two-sample *t*-tests were used to compare means for continuous data (e.g., vehicle speeds) between intervention and control groups. A *p*-value of < 0.05 was considered statistically significant.

## 3 Results

The project yielded significant positive outcomes across all key indicator areas, with updated data from expanded phases confirming and strengthening the initial findings. Throughout both phases, the project successfully executed a wide range of communication and implementation activities, supported by over 32 online news articles, 4 TV news features, 97 billboards, and 160 banners, generating 1,992 entries for a photo contest on Facebook, 105 PSA airings on TV and 600 times on radio to amplify its road safety messages.

### 3.1 Infrastructure safety improvements

Post-intervention assessment utilizing the iRAP Star Rating for Schools (SR4S) protocol confirmed universal safety upgrades across the study area. Prior to modification, the road environments were characterized by critical safety deficits; several zones held ratings as low as 1-star, with the pilot school Phan Dang Luu registering a severe Risk Score of 327.7 (2-star). Following the engineering interventions, 26 out of 27 participating schools achieved a rating of 4 stars or higher, effectively eliminating high-risk infrastructure. Notably, the pilot schools—Phan Dang Luu and Nguyen Luong Bang—improved from 2-star and 3-star baselines to achieve the highest possible 5-star rating, with Phan Dang Luu demonstrating a 98% reduction in its calculated risk score (from 327.7 to 6.2) (Table 1).

**Table 1:** Improvements in iRAP Star Ratings for pedestrian safety at project schools.

#	School name	Pre-modification		Post-modification	
		Star rating	Risk score	Star rating	Risk score
1	Phan Dang Luu	2	327.7	5	6.2
2	Nguyen Luong Bang	3	80.8	5	14.0
3	Nguyen Chi Thanh	1	101.4	5	2.5
4	Le Quy Don	1	132.7	4	10.0
5	Bui Thi Xuan	2	57.5	5	1.0
6	Le Lai	2	77.0	4	7.9
7	Ho Tung Mau	2	78.5	5	0.8
8	Nguyen Duc Canh	2	50.8	4	6.3
9	Tran Quy Cap	3	39.9	5	1.4
10	Chu Van An	3	15.9	5	1.2
11	Nay Der	3	30.1	5	1.2
12	Bui Du	3	18.3	5	0.5
13	Ngo Quyen	3	21.9	5	0.6
14	Nguyen Thi Minh Khai	3	17.9	3	17.3
15	Tran Quoc Toan	3	34.9	5	1.8
16	Le Hong Phong	3	37.6	5	1.4
17	Ngo May	4	14.3	5	0.8
18	Dinh Tien Hoang	4	12.1	5	0.4
19	Nguyen Trai	4	6.6	5	1.8
20	Cu Chinh Lan	4	9.7	5	2.3
21	Luong Thanh	4	6.1	5	0.4
22	Nguyen Van Troi	4	9.7	5	1.7
23	Vo Thi Sau	4	12.8	5	4.2
24	Le Van Tam	4	12.2	5	0.5
25	Hoang Hoa Tham	5	1.8	5	0.4
26	Nguyen Binh Khiem	5	2.4	5	0.3
27	Tran Dai Nghia	5	0.6	5	0.5

### 3.2 Reduced vehicle speeds

A significant reduction in vehicle speeds was consistently observed at intervention schools. In Phase I, independent samples *t*-tests confirmed statistically significant speed reductions at intervention schools. Nguyen Luong Bang Primary School saw a mean reduction of 14.2 km/h (95% CI: 12.3–16.1, *p* < 0.001), and Phan Dang Luu Primary School observed a reduction of 5.4 km/h (95% CI: 3.6–7.2, *p* < 0.01). In contrast, the control school showed no statistically significant change (*p* > 0.05) (Table 2).

**Table 2:** Reduction in mean vehicle speeds (km/h) at school safety zone in Phase I.

School	Mean speed (Pre)	Mean speed (Post)	Diff	95% CI of Diff	*p*-value
Nguyen Luong Bang	49.2	35	−14.2	[−16.1, −12.3]	<0.001
Phan Dang Luu	41.1	35.7	−5.4	[−7.2, −3.6]	<0.01
Anh Hung Nup (control)	42.7	41.5	−1.2	[−3.1, 0.7]	>0.05

The reduction in mean vehicle speeds at the intervention schools was statistically significant compared to the control school (two-sample *t*-test, *p* < 0.01).

**Figure 1:**
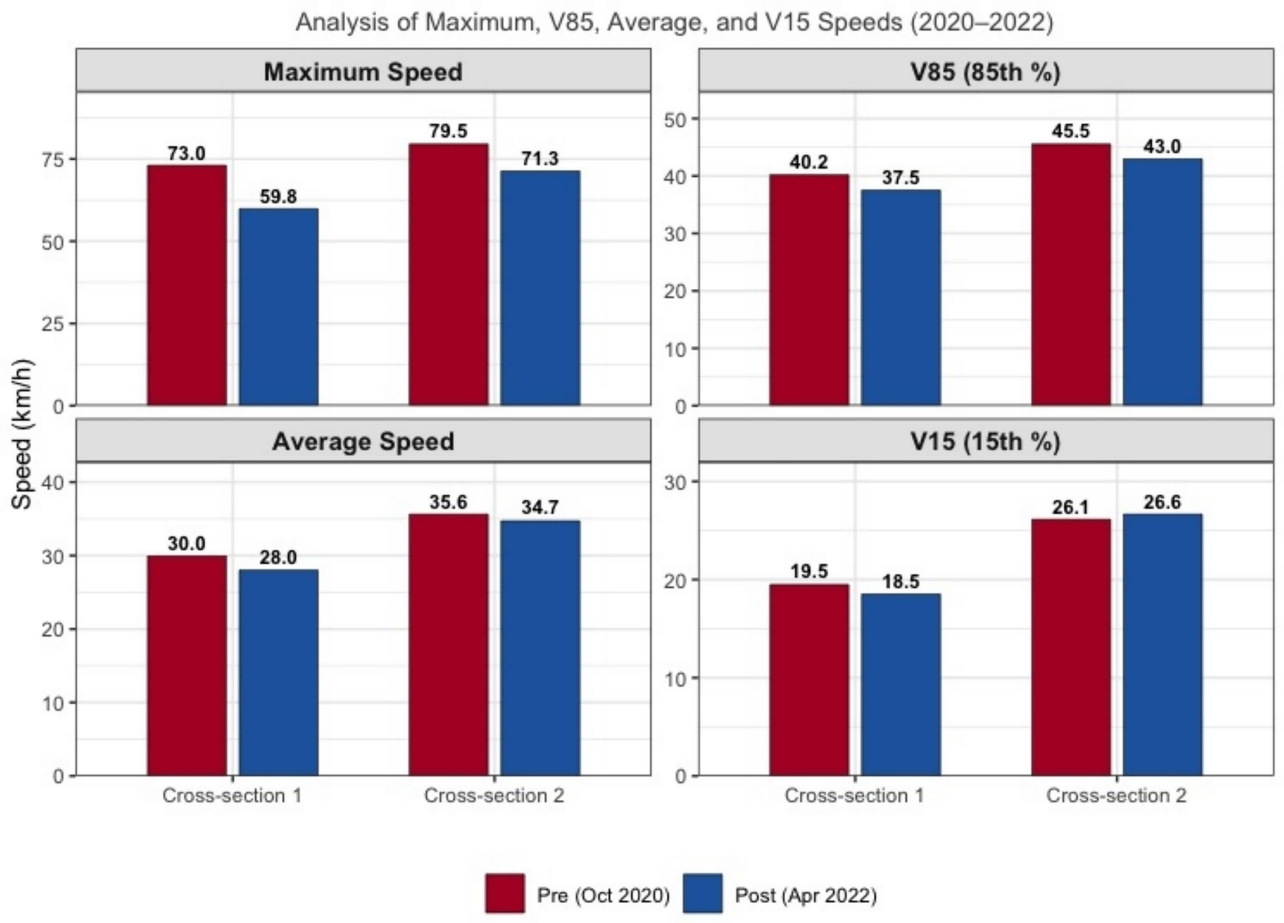
City-wide speed reductions (Phase II).

Figure 1 presents the pre- and post-results for speed measurement of vehicles traveling through the school zones. At the commencement of Phase II in 2020, the observed maximum speed of vehicles passing by the school gates of all surveyed schools was 73 km/h. It was then dropped to 59.8 km/h after 2 years of intervention. The average speed also decreased by 2 km/h in 2022. At cross-section 2, a decrease of 8.2 km/h in the maximum speed was observed, alongside a decrease of 0.9 km/h in the average speed between the pre- and post-intervention. The pre-intervention results indicate that 85% of road users did not exceed the speed of 40.2 km/h (V85 speed) when passing by cross-section 1 and the speed of 45.6 km/h when passing by cross-section 2. In 2022, the V85 speed was reduced by 2.6 km/h for both cross-sections.

### 3.3 Decreased student crash

The combination of safer infrastructure and lower speeds led to a notable reduction in crashes. At the beginning of the project, the students were asked about their crash experiences in the last 12 months and 3 months in the subsequent phase. To account for differences in recall periods (12 months at baseline vs. 3 months at end-line), crash data were standardized as incidence rates per 1,000 student-months. At baseline, the crash rate was 5.49 per 1,000 student-months (95% CI: 4.41–6.73). Following the intervention, this rate declined to 3.84 at the mid-term and further to 2.58 per 1,000 student-months (95% CI: 2.01–3.26) at the end-line, indicating a statistically significant 53% reduction in crash risk post-intervention (*p* < 0.001) (Table 3).

**Table 3:** Crash monitoring results.

Reporting time	Monitoring duration	# surveyed schools	# respondents	# crashes	# crashes in school zones	Crash rate per 1,000 student-month	% crash in school zones
Baseline (April 2018)	12 months	3	1,366	90	32	5.49	35.6%
Mid-term (April 2021)	3 months	10	8,342	96	19	3.84	19.8%
End-line (April 2022)	3 months	10	8,779	68	2	2.58	2.9%

### 3.4 Improved knowledge, attitudes, and practices (KAP)

The project’s education campaigns produced significant improvements in community and student knowledge. Parent awareness of correct speed limits soared from 15.6% at baseline to 93.2% at the endline. Similarly, the percentage of students demonstrating good road safety knowledge increased significantly more in intervention schools (see Table 4).

**Table 4:** Changes in parent road safety knowledge, attitudes, and practices (KAP).

KAP indicator	Baseline	Mid-term	Endline	*p*-value
Aware of speed limits	15.6%	92.2%	93.2%	<0.001
Has good road safety knowledge	n/a	70.2%	71.8%	--
Believe speed reduction is very necessary	88.7%	94.5%	96.5%	<0.001

**Figure 2:**
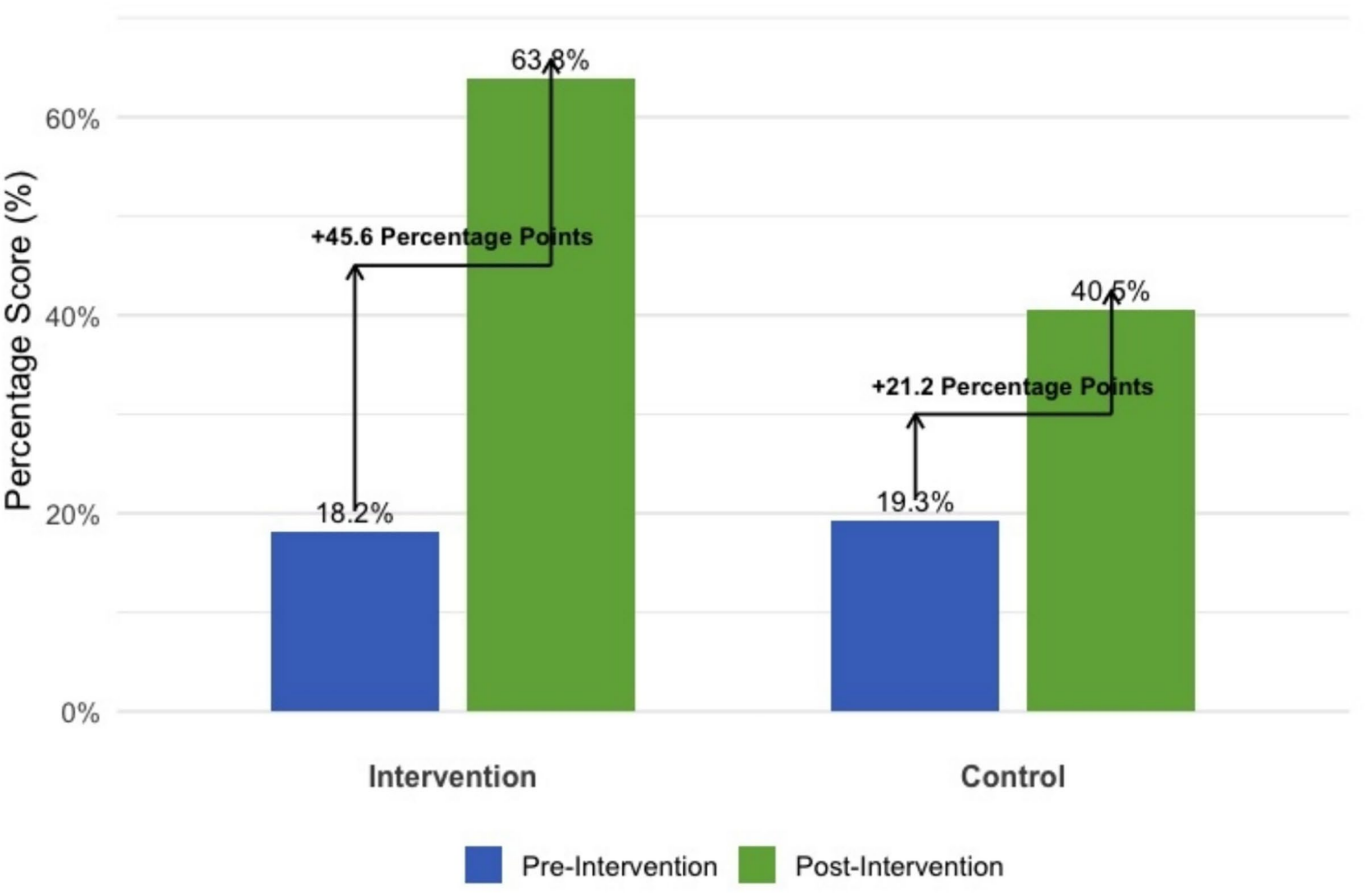
Comparison of improvement in student road safety knowledge.

The increase in parental awareness of speed limits from baseline to endline was statistically significant (Chi-squared test, *p* < 0.001) (see Figure 2).

At baseline, there was no statistically significant difference in road safety knowledge scores between the intervention and control groups (*p* = 0.70). Following the program, paired sample t-tests indicated that both groups achieved a statistically significant increase in knowledge (*p* < 0.001). However, independent sample t-tests on the change scores revealed that the intervention group demonstrated a significantly greater magnitude of improvement (+45.6 percentage points) compared to the control group (+21.2 percentage points) (*p* < 0.001), confirming the specific effectiveness of the intervention beyond general learning effects.

## 4 Discussion

### 4.1 Effectiveness

The results from the SZSZ project demonstrate a clear, significant, and multifaceted positive impact, with its effectiveness firmly rooted in its alignment with the globally recognized ‘Safe System’ approach. This modern road safety philosophy represents a paradigm shift, moving beyond blaming individual road users and instead creating a forgiving transport system that acknowledges human fallibility and manages kinetic energy to survivable levels. The SZSZ project successfully operationalized this paradigm by tackling risk at multiple, synergistic levels.

The attainment of 4- and 5-star ratings across nearly all (97%) project sites provides strong empirical validation that the SZSZ model effectively “engineers out” pedestrian risk, even in complex, motorcycle-dominant traffic environments. By elevating all schools above the global 3-star minimum standard, the intervention aligns directly with the Global Plan for the Decade of Action for Road Safety 2021–2030 (1). These results validate the *Safe System* principle that physical constraints are required to manage kinetic energy in environments where enforcement is intermittent (8). The intervention’s success in reducing risk scores by up to 98% (Phan Dang Luu) aligns with global evidence demonstrating the effectiveness of the iRAP approach. Large-scale analyses show that iRAP-informed road-infrastructure upgrades can prevent hundreds of thousands of deaths and serious injuries worldwide (9).

While the aggregate mean speed reduction of approximately 2.6 km/h in Phase II may appear modest in absolute terms, its public health implications are substantial when interpreted through the ‘Power Model’ of road safety (10–12). This model posits a power function relationship between mean travel speed and crash severity; empirically, a 1% reduction in mean speed correlates with a 4% reduction in the risk of fatal crashes. Consequently, the observed reduction represents a theoretical decrease in fatality risk of approximately 20%, validating the critical impact of the infrastructure modifications. Finally, safer road users were fostered via education, evidenced by the dramatic rise in parental knowledge of correct speed limits from just 15.6% to over 93%. The synergy of these components is validated by systematic reviews which conclude that multi-component interventions yield more significant and sustained safety gains than single-focus initiatives (13). The reduction in vehicle speeds is arguably the most critical outcome, as the laws of physics dictate a pedestrian’s risk of death increases exponentially with speed; the chance of survival is dramatically higher when struck at 30 km/h versus 50 km/h (14).

The project’s outcomes are highly consistent with and build upon findings from international interventions, reinforcing a global consensus on best practices. While research from high-income countries, such as a landmark study in Edmonton, Canada, has quantitatively validated speed reduction as a core safety strategy—showing a 45.3% drop in injury collisions from lowering speed limits alone—the real test of the model’s adaptability is in the complex road environments of LMICs (15). The SZSZ project’s holistic design echoes successful programs across the developing world. For instance, the School Area Road Safety Assessments and Improvements (SARSAI) programme implemented in African cities like Dar es Salaam and Kampala combined infrastructure improvements (e.g., crosswalks, speed humps) with education and reported significant reductions in child pedestrian injury rates (16). Similarly, interventions in several cities in LMICs, under the Bloomberg Philanthropies Initiative for Global Road Safety (BIGRS), demonstrated that traffic calming measures around schools led to significant reductions in vehicle speeds and crashes, reinforcing the importance of the engineering component that was central to the SZSZ project (17).

While consistent with these international successes, the SZSZ project makes two particularly vital contributions to the global evidence base. First, it demonstrates the model’s profound effectiveness in a traffic environment heavily dominated by motorcycles, a chaotic and under-studied context that presents unique challenges not found in the car-dominant settings of many other studies. By succeeding in this environment, the project confirms that the Safe System approach is robust and adaptable across diverse traffic mixes when tailored to local needs. Second, the project successfully created a “policy feedback loop.” The strong commitment of Pleiku’s local authorities, national stakeholders such as the National Traffic Safety Committee and the Ministry of Education and Training, together with funders (i.e., Foundation Botnar and FIA Foundation), non-profits, and the private sectors, formed a powerful coalition for change. While other interventions have demonstrated local success, the SZSZ project provides a clear blueprint for how to leverage credible, internationally recognized evaluation data—specifically from the iRAP SR4S tool—to secure government buy-in and catalyze systemic, nationwide change. This evidence was the critical advocacy tool that led to legislative change, sustainable government funding, and ultimately, the development of the national Safe School Zones Vietnam Guide. This successful translation of local evidence into national policy offers a powerful and replicable model for other LMICs aiming to institutionalize child road safety and move beyond isolated pilot projects to achieve lasting, scaled-up impact. One of the most critical achievements of this project was the passage of the first legal document, regulating speed limits of 30–40 km/h around school zones in Pleiku City. A distinct strength of the SZSZ model is its demonstrated cost-effectiveness and transferability. The intervention cost was estimated at approximately $10,000–$15,000 USD per school. The project demonstrates that such gains are achievable through low-cost countermeasures—such as speed humps and raised crosswalks—which have been proven to yield high safety returns in comparable LMIC settings. For instance, recent evaluations of school zone interventions in Addis Ababa (2024) and the SARSAI programme in Tanzania (2019) confirmed that similar infrastructure treatments reduced operating speeds by over 25% and injury rates by 26%, with implementation costs as low as $10,000–$25,000 per site (16, 18).

Additionally, the city officially adopted a School Zone Definition to guide local implementation. Building on the success of the Pleiku project, the Safe School Zone (SSZ) Guide was later developed, using Pleiku’s experience as a model for broader application across Vietnam.

### 4.2 Limitations

This study has several limitations. First, the design shifted from a quasi-experimental model to a city-wide pre-post evaluation. While limiting internal validity regarding external confounders, this was an ethical imperative to scale safety interventions to all schools rather than maintaining a control group. Second, the transition from handheld radar to video-based monitoring improved data quality but introduced instrumentation variance, affecting longitudinal comparability. Third, reliance on self-reported crash data introduced recall bias, which was mitigated by shortening the recall period to 3 months and standardizing analysis via incidence density rates. Finally, despite COVID-19 lockdowns typically encouraging higher vehicle speeds due to reduced congestion, this study observed sustained reductions, suggesting infrastructure robustness. Findings reflect an urban context and may require adaptation for rural settings.

### 4.3 Policy implications

The SZSZ project offers critical lessons for future road safety initiatives. First, it demonstrates the economic viability of the Safe System approach in LMICs. With an estimated implementation cost of approximately $10,000–$15,000 USD per school, this model presents a fiscally sustainable investment for governments compared to high-cost infrastructure overhauls.

Second, the project highlights the necessity of systemic institutionalization over temporary donor reliance. The successful transfer of financial responsibility to city authorities—who have integrated school zone upgrades into municipal public works budgets—provides a blueprint for long-term sustainability. While this integration subjects safety upgrades to government fiscal cycles, it signifies a deeper, more permanent commitment than ad-hoc pilot funding.

Finally, the project establishes a proven “policy feedback loop.” The passage of Pleiku City’s first legal document regulating speed limits of 30–40 km/h, along with the adoption of the School Zone Definition and the creation of the *National Safe School Zone Guide*, are monumental achievements. This evidence-based advocacy demonstrates that local interventions can serve as catalysts for national policy reform, breaking regulatory inertia to create a sustainable, top-down framework that institutionalizes safer school zones across the entire country.

## 5 Conclusion

This evaluation provides robust empirical evidence that the ‘Slow Zones, Safe Zones’ intervention successfully mitigated road traffic risks for children in Pleiku City. By operationalizing the Safe System approach through synergistic infrastructure, enforcement, and education strategies, the project achieved statistically significant reductions in vehicle speeds and crash incidence, while elevating school zone safety ratings to international standards. Beyond immediate safety gains, the project’s most enduring contribution is its demonstration of a ‘policy feedback loop,’ where local pilot data successfully catalyzed legislative change and government institutionalization. This scalable model offers a validated pathway for other low- and middle-income countries to bridge the gap between ad-hoc interventions and national policy reform, directly advancing the targets of SDG 3.6.

## Data Availability

The raw data supporting the conclusions of this article will be made available by the authors, without undue reservation.
